# Measurable Residual Disease in Acute Myeloid Leukemia Using Flow Cytometry: A Review of Where We Are and Where We Are Going

**DOI:** 10.3390/jcm9061714

**Published:** 2020-06-03

**Authors:** Caroline Dix, Tsun-Ho Lo, Georgina Clark, Edward Abadir

**Affiliations:** 1 Institute of Haematology, Royal Prince Alfred Hospital, Camperdown, NSW 2050, Australia; 2 Dendritic Cell Research, ANZAC Research Institute, Concord, NSW 2139, Australia; kevthlo@gmail.com (T.-H.L.); georgina.clark@sydney.edu.au (G.C.); 3 Immunology, Sydpath, St Vincent’s Hospital, Darlinghurst, NSW 2010, Australia; 4 Faculty of Medicine and Health, The University of Sydney, Camperdown, NSW 2039, Australia

**Keywords:** Acute myeloid leukemia, measurable residual disease (MRD), immunophenotype

## Abstract

The detection of measurable residual disease (MRD) has become a key investigation that plays a role in the prognostication and management of several hematologic malignancies. Acute myeloid leukemia (AML) is the most common acute leukemia in adults and the role of MRD in AML is still emerging. Prognostic markers are complex, largely based upon genetic and cytogenetic aberrations. MRD is now being incorporated into prognostic models and is a powerful predictor of relapse. While PCR-based MRD methods are sensitive and specific, many patients do not have an identifiable molecular marker. Immunophenotypic MRD methods using multiparametric flow cytometry (MFC) are widely applicable, and are based on the identification of surface marker combinations that are present on leukemic cells but not normal hematopoietic cells. Current techniques include a “different from normal” and/or a “leukemia-associated immunophenotype” approach. Limitations of MFC-based MRD analyses include the lack of standardization, the reliance on a high-quality marrow aspirate, and variable sensitivity. Emerging techniques that look to improve the detection of leukemic cells use dimensional reduction analysis, incorporating more leukemia specific markers and identifying leukemic stem cells. This review will discuss current methods together with new and emerging techniques to determine the role of MFC MRD analysis.

## 1. Introduction

Acute myeloid leukemia (AML) is a hematologic malignancy characterized by more than 20% blasts in the bone marrow and often with recurring genetic abnormalities. Diagnosis is based on morphology, confirming immunophenotype by flow cytometry, and detection of genetic abnormalities, some of which are diagnostic even in the absence of 20% blasts. Remission status is similarly based on morphology, confirmed with the finding of <5% blasts remaining in the bone marrow following treatment. Prognosis in AML is dependent on both patient and disease factors, with significant weight placed on genetic and cytogenetic features. These features allow for categorization of patients as having adverse-, intermediate-, or favorable-risk as outlined by the European LeukemiaNet (ELN) criteria [1]. However, despite improvements in the characterization, prognostication and treatment of AML, approximately 50% of patients will relapse after initial therapy [2]. 

Measurable residual disease (MRD) detection is an emerging prognostic marker used extensively in acute lymphoblastic leukemia (ALL), particularly in the pediatric setting, and has resulted in improved survival and reduced morbidity [3,4]. MRD denotes the presence of leukemia cells at frequencies below that of routine measurement by morphology or cytogenetics, with sensitivity down to 1 in 10^4^ to 1 in 10^6^ of total leukocytes, as compared to 1 in 20 in standard morphology [5]. Numerous studies have shown the power of MRD in AML in predicting risk of relapse and overall survival [6,7,8,9]. In the setting of allogeneic stem cell transplant (alloSCT), those who proceed to transplant with MRD positivity have been shown to have a risk of relapse and 3-year overall survival similar to those with active AML [10]. Prognostic criteria have now expanded to suggest inclusion of MRD as an outcome measure. Assigning every patient an MRD profile as recommended by the ELN, acknowledges the important role of MRD as a post-diagnosis prognostic marker and its subsequent implications in decision-making [5].

Despite clear evidence that MRD positivity at various timepoints in AML treatment predicts relapse, less is known about how this information will result in different treatment pathways. MRD is currently being used to guide treatment pathways in pediatric and young adult ALL as standard of care, and is a possible predictor of the way MRD assessment can be integrated into treatment decisions for AML in the future. In ALL, those who are MRD negative complete high-dose chemotherapy alone, whereas those who have high levels (≥1%) MRD at the end of induction or persistent low level MRD have their treatment intensified and are usually recommended to proceed to alloSCT if a donor is available [11]. 

Modalities for detecting MRD include real time quantitative polymerase chain reaction (qPCR), next generation sequencing (NGS), and multiparametric flow cytometry (MFC). qPCR methodologies are based upon the detection of the aberrant mutation in leukemic cells as compared to a housekeeper gene, and are sensitive down to frequencies of 1 × 10^6–7^, however this varies significantly depending on the genetic target [1]. While this is highly sensitive, only 60% of younger patients, and fewer older patients, have an identifiable PCR target. Immunophenotyping by MFC to assess MRD is applicable to most patients and is based on the identification of surface marker combinations that are present on leukemic cells but not normal hematopoietic stem cells [12]. There are two major approaches for evaluating MFC MRD. Leukemia associated immunophenotype (LAIP) defines individual-specific surface makers at diagnosis and tracks these in subsequent assessments [12]. The different from normal (DfN) approach is based on the identification of aberrant surface marker profiles at follow-up, can be applied in the absence of diagnostic material, and is able to identify immunophenotype shifts [13]. Limitations of MFC for MRD include a lack of standardization, reliance on a high-quality marrow sample, as well as limitations on sensitivity and gaps in data analysis [14]. New techniques to overcome this include integrating a wider range of surface markers with improved specificity for residual leukemic cells, the use of an algorithmic approach to assign MRD status, and evaluation of leukemic stem cells (LSCs).

This review will discuss the use of MFC in the diagnosis of AML; the role of, and evidence for, MRD in AML; and in particular the use of MFC in MRD in AML; and the new and emerging methods for assessing MRD by MFC.

## 2. Use of Flow Cytometry in Diagnosis

Traditionally, immunophenotyping by MFC has used cluster of differentiation (CD)-lineage specific antibodies to identify the lineage of an acute leukemia. While the current World Health Organization (WHO) criteria for blast enumeration is by morphology, immunophenotyping can assist in difficult cases, particularly in distinguishing normal from abnormal progenitors [15,16]. The principle of immunophenotyping in AML relies on the knowledge of the tight control of the maturation and differentiation of normal hematopoietic cells with consistent expression of cell surface molecules at defined stages of maturation; neoplastic cells lose this regulation which results in changes in molecule expression [15]. 

Characterization of acute leukemias often starts by gating of CD45 against side scatter, to identify myeloid blasts, which are commonly CD45 dim and often with high side scatter, depending on their granularity [16]. This is opposed to lymphoblasts, which are most commonly dim-to-negative for CD45 with low side scatter. The immature nature of the blasts is confirmed by CD34, CD117, and HLA-DR positivity; further lineage specification is established by CD13 and CD33 confirming myeloid maturation, and myeloperoxidase (MPO) and CD15 neutrophilic lineage. There may be aberrant lymphoid markers such as CD7 or CD19 present. These are useful to confirm that the blasts are from the same clone rather than ‘normal’ regenerating blasts (particularly after initial chemotherapy). 

Immunophenotyping can be used to correlate to the WHO category of AML. For example, AML with *RUNX1-RUNX1T1* (t(8;21)) shows a classical expression of CD34 and CD117, alongside CD13, CD33, and MPO, as well as evidence of neutrophilic maturation with some expression of CD15 and/or CD65; often there is aberrant expression of CD19 [17]. Acute promyelocytic leukemia (APL) with *PML-RARA* (t(15;17)) usually have high side scatter, and express typical myeloid markers of CD13, CD33, and CD117, with strong MPO but lack CD34 [18]. AML with *CBF-MYH11* (inv(16)) have a typical myeloid immunophenotype but often with monocytic markers such as CD4, CD36, or CD38 [19]. AML with nucleophosmin 1(NPM1) can have myeloblastic or monoblastic differentiation, but frequently do not express CD34 and/or HLA-DR [20].

Certain markers have been purported to be associated with poorer prognosis. Expression of CD7, CD9, CD11b, CD13, CD14, CD33, CD34, CD56, and terminal deoxynucleotidyl transferase (TdT) have all been associated with poor prognosis; co-expression of CD34 and HLA-DR is an independent predictor of failure to achieve complete remission [17]. Expression of pan-myeloid markers is associated with a more favorable prognosis [21].

## 3. Current Methods of Assessing Measurable Residual Disease

Two major approaches are used to detect MRD: MFC and molecular techniques. Currently, there is no standardization about which time point should be used to assess MRD, and different times may give different information—early assessment (following induction and/or consolidation) can be used to assess remission status and determine the kinetics of the disease response; later assessments can be used to identify impending relapse. Table 1 outlines a comparison of the techniques used for MRD assessment.

### 3.1. Molecular Methods

Approximately 60% of patients with AML will have a molecular marker suitable for validated qPCR-based MRD analysis, and this number is increasing with the identification of new molecular targets [5]. Approximately 15–35% of patients with AML harbor a gene fusion that can be tracked with qPCR such as *RUNX1-RUNX1T1* (t(8;21)), *CBF-MYH11* (inv(16)), and *PML-RARA* (t(15;17)) [18]. Another 25% have an *NPM1* mutation [18,30,31]. The assays have been standardized and are based on the relative expression of the mutation target compared to a standard housekeeping gene (often ABL1) in leukemic blasts. The *NPM1* mutation assays have sensitivities up to 1 in 10^6–7^ due to increased expression of the mutant allele; other assays are less sensitive due to varying expression of the molecular marker [24,25,32]. MRD response and relapse kinetics are also quite different depending on the target. The time from molecular positivity to clinical relapse in inv(16) is rapid at 3-months, and for t(8;21) it is slightly longer at ~4.5-months [25]. Interestingly, patients with t(8;21) can harbor low levels of MRD and maintain a durable remission and thus MRD negativity is not a prerequisite for long-term remission with this rearrangement [25]. Current ELN consensus is to monitor these mutations at diagnosis, after two cycles of induction/consolidation therapy and every 3-months for 24-months after the end of treatment [1]. 

NGS is becoming a standard assessment at diagnosis to enable further risk stratification, particularly in two patient cohorts [33]. The first is for those who are potential candidates for treatment intensification. The second cohort is patients who have cytogenetically normal AML, which is known to have significant mutation heterogeneity but have been clustered together due to difficulties in further routine sub-stratification [33]. The role of NGS for MRD assessment is hindered by the presence of clonal hematopoiesis, with some common persisting mutations (*DNMT3A*, *ASXL1,* and *TET2*) not having any prognostic role [26]. NGS techniques have enhanced our understanding of clonal evolution, in particular for mutations such as FMS-like tyrosine kinase 3 internal tandem duplication (*FLT3*-ITD) which cannot be used as an MRD marker because ~25% of patients relapse with *FLT3*-ITD negative disease [34]. While NGS can be highly sensitive (>1 × 10^−6^), it lacks standardization, requires a high degree of expertise to interpret, and is currently expensive [27]. NGS is also limited by sequencing error rates of approximately 1% which reduces its sensitivity due to a difficulty in distinguishing true mutations with a low variant allele frequency from sequencing errors [28]. 

Newer molecular methods being evaluated include digital droplet PCR, currently used in the diagnostic molecular hematopathology space, which is highly sensitive, specific, and allows for an absolute quantitation (as opposed to qPCR which is relative); and targeted RNA sequencing which can have a sensitivity of 1 in 10^5^, although there is significant inter-individual differences in RNA expression limiting sensitivity [35,36].

### 3.2. Flow Cytometric Methods 

Detection of MRD with MFC uses similar principles to diagnostic immunophenotyping but requires more sophisticated antibody combinations to detect the very low frequency of leukemic cells within an otherwise normal bone marrow [22]. MRD using MFC is based on the detection or combination of cell surface markers present on leukemic blasts but not on normal hematopoietic stem cells. MFC has the advantage over PCR methods that it is applicable to over 90% of AML patients and is rapid, potentially allowing real-time therapeutic decision-making [23]. Two varying approaches are that of LAIP or DfN. LAIP identifies a unique immunophenotype at diagnosis and follows it over time [37]. This approach assumes the stability of the immunophenotype after therapy, and it is known that they can, and do, change [22]. In the DfN approach, a standardized combination of antibodies is applied to all MRD analyses, regardless of the diagnostic LAIP, allowing for identification of markers outside the spectrum of the maturation process of normal stem cells [36]. DfN can still be applied without knowledge of the diagnostic LAIP, however is more difficult to standardize due to inherent subjectivity in interpretation [38]. While it is difficult to compare methods directly due to differences in patient cohorts, treatments given and cutoffs used, DfN may have a lower sensitivity compared to LAIP, with a trial using the DfN approach finding a lower percentage of MRD-positive patients in complete remission (CR; 24%) [39], compared to those using LAIP (34–42%%) [40,41]. However, due to the phenotypic shifts that often occur at relapse in AML, the use of LAIP may increase false negativity if an extended antibody panel is not used [38]. Sensitivity of MFC by both methods is variable, and is influenced by the number of cells defining the leukemic population, the total number of cells evaluated, the gating method, and the number of antibody colors used, but is generally around 1 in 10^3^ to 10^5^ [10,38,42,43].

Current ELN guidelines on MRD in AML recommends harmonization of these approaches, with a minimum of eight fluorochromes and using the same tubes (with the same antibody-fluorochrome combinations) at diagnosis and at follow up, to allow for the tracking of both the LAIP established at diagnosis and for emerging phenotypic shifts [5]. They propose gating on CD45, side scatter, forward scatter, a primitive marker (CD34, CD117), and abnormal expression of marker/s or combination/s of marker expression including CD7, CD11b, CD13, CD15, CD19, CD33, CD56, and HLA-DR; a monocytic tube (CD64, CD11b, CD14, CD4, CD34, HLA-DR, CD33, and CD45) is recommended for monocytic or myelomonocytic AML [5]. Between 500,000 and 1 million viable cells are required to obtain the recommended 0.1% as the cutoff between MRD positive from MRD negative; this is despite acknowledgement that MRD quantification below 0.1% may still represent residual leukemia [5]. In order to obtain an adequate sample, the first bone marrow pull should be used with minimal blood dilution and be processed as soon as practicable after collection [23]. Only operators with extensive experience in MRD interpretation should do so, and it is important to use published protocols [23]. 

There are several limitations to the use of MFC for MRD. There are still ~25% of patients who relapse despite achieving MRD negativity [44]. Both technical and biological reasons may account for this. Technically, reproducibility remains a major concern, largely due to a lack of standardization of assays between laboratories, such as differing surface markers used and varying levels used to define positivity and negativity, and the inherent need for a subjective interpretive component to MFC analysis, although the ELN consensus recommendation is hoping to improve this [5,22,38,40]. Poor quality marrow samples, with significant hemodilution, can interfere with the specificity of the MFC analysis and also reduce its sensitivity [36]. Biologically, relapse of MRD negative patients may be due to the presence of LSCs which are undetectable by routine methods; ELN recommends the inclusion of a tube to determine residual LSCs in MFC MRD analysis [5].

## 4. Role of MRD Assessment in Acute Myeloid Leukaemia 

Current methods for assessing remission status are based on morphology, flow cytometry and, in some cases, cytogenetics. Most therapeutic decision-making until now has been based on morphological and cytogenetic assessment, however they are crude measures of remission, with poor sensitivity and significant inter-observer variation. Even with the incorporation of prognostic cytogenetic and molecular markers into therapeutic decision-making, only 35–40% of patients under 60 years, and 5–15% of those older than 60 years are cured [45]. Establishing a more precise measure of residual disease is where the incorporation of MRD is key. As a post-diagnosis prognostic marker, MRD can objectively define a deeper remission status, refine outcome prediction, and in some circumstances, inform post-remission treatment (i.e., alloSCT vs. consolidation chemotherapy), as well as identify impending relapse, enabling early intervention and robust post-transplant surveillance [5]. MRD is becoming systematically incorporated into decision making for alloSCT in patients who are in first CR and its role is predicted to expand [46]. 

The evidence of the value of MRD in predicting relapse is convincing. Monitoring of *PML-RARA* transcripts has been standard of care in APL for many years, and early intervention at the time of molecular relapse improves survival compared to frank relapse [47,48]. Such guidelines in non-APL AML are not as clear, particularly regarding management of molecular relapse. Classically, patients with a “favorable risk” genetic marker (*RUNX1-RUNX1T1*, *CBF-MYH11* or *NPM1*) have been treated with chemotherapy alone, and only proceeded to transplant in certain circumstances. Despite their favorable risk status, these patients can still relapse and further refinement of risk within this category has therefore been examined. For example, the persistence of NPM1-mutated transcripts following the second chemotherapy cycle is associated with a significantly higher risk of relapse at 3-years than the absence (82% vs. 30%) and a lower rate of survival (24% vs. 75%) [9]. 

Clinical outcomes based on MRD status using MFC has been evaluated and are outlined in Table 2. A prospective analysis of MRD using LAIP in the HOVON/SAKK (Dutch-Belgian Haemato-Oncology Cooperative Group and the Swiss Group for Clinical Cancer Research) AML42a study found clear correlation between MRD status and outcome [42]. They found that MRD positivity after cycle 2 to be associated with a higher risk of relapse, with 4-year relapse-free survival (RFS) of 23% and relapse incidence of 72% compared to 52% and 42%, respectively, for MRD negative patients. MRD was predictive, independent of other prognostic markers and in particular in the ELN intermediate-risk group where treatment decisions can be difficult. In an effort to establish MRD status very early in treatment, which would enable treatment intensification as soon as possible, Kohnke et al. assessed the prognostic value of MFC (LAIP) MRD during aplasia on day 16–18 of induction therapy [49]. They found that flow MRD positivity at this time point independently predicted poor outcome, with a 5-year RFS of 16% compared to 43% for MRD negative.

MRD by MFC in those who do not harbor an appropriate molecular target has been shown to be useful in therapy decisions. The GIMEMA (Gruppo Italiano Malattie EMatologiche dell’Adulto) young adult AML1310 trial showed that while intermediate-risk patients with MRD negativity could avoid an alloSCT, those who were MRD positive could have a prolonged survival and relapse risk similar to those in the MRD negative group with an alloSCT [50]. While current ELN recommendations are to use MFC only if there is no standardized molecular target, combining modalities is likely to increase sensitivity [5,26,29]. Multiple studies comparing MFC to molecular methods (both qPCR and NGS) have found concordance of approximately 70% [26,29,42,50]. In the setting of NGS, this discordance may be due to presence of residual leukemia mutations with the variant allele frequency below the threshold for mutation calling, typically 5% [29]. These studies have shown concurrent MRD negativity by MFC and NGS/qPCR had the lowest relapse rate, concurrent MRD positivity had the highest relapse rate, and discordant MRD results were in between [26,29,42,54]. 

MRD allows for prognostication and decision-making in the setting of alloSCT. MRD negativity using a 3 tube, 10 color flow cytometry panel, confers favorable outcomes (3-year overall survival (OS) estimates of >70% and a relapse risk of 20–25%) after myeloablative alloSCT [10]. This is compared to patients in morphologic remission but with MRD positivity, who have an OS of only 25% and relapse risk of ~70%, which is similar to those who enter transplant with morphologically detectable disease [10]. Conversion from MRD positivity pre-transplant to MRD negativity after myeloablative conditioning may not substantially improve relapse rate or survival [51]. Further stratification along a continuum of MRD levels rather than binary positive/negative may be beneficial, with a recent study of outcomes of alloSCT using pre-transplant MRD in *NPM1* positive patients finding only those with high MRD levels and those with concomitant *FLT3*-ITD having poor outcomes (as opposed to MRD negative or low) [55].

It is clear that MRD assessment in those in morphologic CR can identify those at high risk of relapse. Ideally, this information would then be used to dictate those who require treatment intensification, or pre-emptive therapy at molecular (as opposed to hematologic) relapse, as it has been done with ALL and APL. In the setting of alloSCT, identification of persistent MRD positivity or patients becoming MRD positive after previously being negative, may allow pre-emptive therapy, for example rapid weaning of graft-versus-host disease (GVHD) prophylaxis or donor lymphocyte infusions (DLI) [56,57]. Treatment with azacitidine based on MRD positivity after alloSCT has shown promise, but larger prospective studies are needed [58,59]. In addition, MRD may be able to be used as a surrogate survival end-point for clinical trials, allowing for faster drug approval [60]. There are ongoing issues impeding the routine use of MRD in AML, in particular how to use the information to improve outcomes, incorporating results into prognostic scores and ultimately management options. It is not clear regarding at what time point MRD should be evaluated; studies so far have evaluated MRD at various time points (post-induction vs. post-consolidation). While risk-adapted therapy has started to be evaluated it is still not clear regarding management of high-risk patients or whether therapy at molecular relapse will have better outcomes than waiting for hematological relapse [50,61]. MRD assessment is often not warranted for older individuals who would not be candidates for treatment intensification, although this may change in the advent of newer targeted therapies [43]. In addition, until MRD assessment becomes more available and standardized, particularly MFC, the choice of modality will be based on availability rather than gold-standard, and even this has yet to be refined.

## 5. New and Emerging Strategies in Multiparametric Flow Cytometry

Despite evidence for the key role of MRD analysis in the post-diagnostic assessment of patients with AML and its power in predicting relapse, there remain patients that relapse despite being MRD negative as assessed by PCR methods and/or MFC. The limitations of current methods of MRD detection necessitate improvement. 

Assessment of LSCs improves the prognostic impact of MRD (using both MFC and PCR methods) detection [52]. LSCs are defined as cells that are capable of initiating disease and have the capacity to self-renew; it is thought that to eradicate leukemia, these LSC populations must be eliminated [62]. Small subpopulations of LSCs may be more therapy-resistant than the bulk of leukemia cells and form the basis of relapses. These LSCs can be CD34^+^CD38^+^, CD34^+^CD38^−^, or CD34^−^, although it is thought the CD34^+^CD38^−^ population are the most therapy-resistant and increased numbers are associated with an adverse prognosis [63]. Including CD34^+^CD38^-^ LSCs in standard MFC MRD analysis enhanced the assessment and further teased out prognostic value after second induction: those who were MRDneg and LSCneg (CD34^+^CD38^−^) had the best prognosis (3-year cumulative incidence of relapse (CIR) 35% and 3-year overall survival 66%), while those who were MRDneg but LSCpos with a CIR of 53% and OS of 53%, highlighting the added information LSC interrogation brings to the analysis [52]. Those who were MRDpos and LSCneg had a similar CIR to both being negative (CIR 43% and 3-year OS 68%), while MRDpos and LSCpos had a dismal prognosis with 100% relapsing within 3 years [52]. Adding CD123 to CD34^+^CD38^−^ has been shown to be highly sensitive and specific to leukemic cells. With CD123 being present on 97% of AML CD34^+^CD38^−^ stem cell populations, but not present on either normal or regenerating bone marrow, it can specifically discriminate leukemic cells [64]. The addition of other markers, such as CLL1, CD44, GPR56, and CD184, may provide further improvement in LSC specificity and prognostic information [43,65].

New targets to differentiate leukemic blasts from normal cells will enable more sensitive MRD analysis. These include ILT3 (CD85k) which is expressed in AML with monocytic differentiation and has high sensitivity and stable expression [66]. There are ongoing studies into AML immunotherapy which have identified a number of promising makers that differentiate leukemic cells from healthy hematopoietic stem and progenitor cells (HSPC), which could be incorporated into AML MFC MRD analysis [67]. Genome-wide studies analyzing expression of AML cells compared to normal myeloblasts have discovered an abundance of aberrantly expressed markers (CD9, CD18, CD25, CD32, CD44, CD47, CD52, CD54, CD59, CD64, CD68, CD86, CD93, CD96, CD97, CD99, CD123, CD200, CD300a/c, CD366, CD371, and CX3CR1) [53]. While additional markers may increase sensitivity (in the Coustan-Smith study, down to one leukemic cell in 10^5^ normal cells) and specificity of MRD testing, and allow MRD analysis in patients who may not have an LAIP, a major issue is data interrogation. Machine-learning algorithms, such as t-distributed stochastic neighbor embedding (tSNE), which visualizes high-dimensional data by giving each datapoint a location in a 2-D or 3-D map, will enable easier analysis of the huge amounts of data being generated [68]. Machine-learning algorithms in this context significantly reduce interpretation time (7 seconds using algorithms versus at least 20 minutes manually for an experienced hematologist) and reduce the potential for bias and other subjective errors, which can be a major flaw in MRD by MFC [69]. 

Mass cytometry time of flight (CyTOF) involves labelling antibodies with heavy metal isotopes instead of fluorochromes and evaluating samples using time-of-flight mass spectrometry [70]. This approach has many advantages. CyTOF allows a larger number of parameters (greater than 40) to be evaluated and eliminates much of the “background noise” which can be a problem in high sensitivity MFC assays [36]. This is very well suited to assessing MRD, due to the markedly heterogeneous cell populations in AML, both within and between individuals at different time points. Gjertsen et al. also argue that it may allow for earlier assessment of MRD and a personalized optimization of therapy. However, its more widespread use is limited by the need for complex analytical tools, lack of validation in large clinical trials and expense [36,70].

## 6. Conclusions

AML is a highly heterogenous malignancy and prognosis has been based around patient and disease factors, with a major emphasis on recurring genetic abnormalities. However, despite improved understanding on the biology and kinetics of the disease response to treatment, many patients relapse despite achieving an initial remission, and thus improved methodologies for remission status have been required. MRD is rapidly becoming the most powerful predictor of relapse in AML. Multiple studies have shown those who are (or remain) MRD positive at various time points in treatment have a much higher risk of relapse and lower overall survival. As MRD assessment becomes standard of care in AML, understanding the methods available and appropriateness to each patient will be imperative in correctly interpreting the results. PCR-based methods are highly sensitive and applicable to approximately 60% of patients using qPCR; the emerging role of NGS and digital droplet PCR are further being defined. MFC is more widely applicable but has inherently more subjectivity in its interpretation. The ELN consensus recommendations for the use of MFC for MRD assessment are a great step in improving standardization in the use of MFC. The current limitations of MFC for MRD assessment are being addressed by different approaches. New methods of data interrogation, integration of novel markers, and further refinement of the LSC population allow for ongoing improvement in sensitivity and applicability. Further research into incorporation of MRD into treatment algorithms and benefit of pre-emptive intervention are required.

## Figures and Tables

**Table 1:** Comparison of methods used for measurable residual disease (MRD) assessment.

MRD Methodology	Sensitivity	Advantages	Disadvantages	References
MFC-LAIP	1 in 10^3^–10^5^	Sensitive Widely available technology Applicable to >90% of patients Rapid turnaround time	Requires diagnostic sample Extended antibody panel required Does not take into account phenotypic shifts Limited standardization	[5,13,14,15,22]
MFC-DfN	1 in 10^3^–10^5^	Sensitive No diagnostic sample required Phenotypic shifts will not interfere with results Applicable to >90% of patients Rapid turnaround time	Significant operator experience required Limited standardization	[5,14,15,22,23]
qPCR	1 in 10^4^–10^6^	Sensitive Standardized	Appropriate targets present in only approximately 60% of patients Many mutations not suitable for MRD (such as FLT3) Time consuming	[5,9,24,25]
NGS	Variable	Potentially highly sensitive Multiple mutations detected/followed at once	Confounded by pre-leukemic mutations Time consuming Technology not widely available Error rates Expensive Not standardized Complex interpretation	[26,27,28,29]

LAIP = leukaemia-associated immunophenotype; DfN = different from normal; qPCR = real time quantitative polymerase chain reaction; NGS = next generation sequencing; FLT3 = FMS-like tyrosine kinase 3.

**Table 2:** Comparison of strategies used for MRD detection using multiparametric flow cytometry (MFC) and their clinical evidence.

Reference	MFC Method	Methodology/Surface Markers Used	Time Point of Assessment	Evidence-Outcomes
[42]	MFC-LAIP	Limit of detection 0.1%	After induction therapy	MRDneg: median RFS >47 months; 4-year RFS 52% MRDpos: median RFS 8.6 months; 4-year RFS 23%
[50]	MFC-LAIP combined with qPCR	8 color MFC assay	After consolidation	Both neg: 2-year OS 89% and DFS 69% MFCpos/PCRneg or MFCneg/PCR pos: 2-year OS 88–89%; DFS 65–76% Both pos: 2-year OS 55%, DFS 22%
[26]	MFC-LAIP combined with NGS	Limit of detection 0.1%	After induction therapy	Both pos: 4-year relapse rate 73.3% NGSpos/MFCneg: 4-year relapse 52.3% NGSneg/MFCpos: 4-year relapse 49.8% Both neg: 4-year relapse rate 26.7%
[49]	LAIP at time of bone marrow aplasia (day 16–18)	31 surface markers, multiple LAIPs identified for each patient Limit of detection 0.15%	Day 16–18 of induction therapy	MRDneg: 5-year RFS 43% MRDpos: 5-year RFS 16%
[10]	MFC-DfN	10 color assay 3 tubes, 1 million events per tube. Limit of detection 0.1%	MRD assessment pre-alloSCT and outcomes post-transplant	MRDneg: 3-year OS >70%; relapse risk 20–25% MRDpos: 3-year OS 25%, relapse risk 70%
[51]	MFC-DfN	10 color MFC assay Surface markers: CD4, CD5, CD7, CD13, CD14, CD15, CD16, CD19, CD33, CD34, CD38, CD45, CD56, CD64, CD71, CD117, CD123, HLA-DR. Any measurable MRD considered positive	Pre-alloSCT and post-alloSCT (day +28)	MRDpos pre-alloSCT and neg post: 3-year OS 29% and RFS 18% MRDpos both timepoints: 3-year OS 19%, 3-year RFS 14% MRDneg at both time points: OS 76%, RFS 71%
[29]	MFC-DfN separately and combined with NGS	10 color MFC assay Limit of detection 0.1%	Pre-alloSCT	MFC–MRDpos: 18-month relapse incidence 37%; OS 48% MFC–MRDneg: 18-month relapse 9%; OS 73% Both MFC and NGS pos: 18-month relapse 51%; OS 51% Both MFC and NGS neg: 18-month relapse 8%; OS 78% MFCneg/NGSpos or MFCpos/NGSneg: 18-month relapse 17%; OS 44%
[52]	Combined MFC-LAIP and LSC	Limit of detection 0.1% LSCs defined as CD34^+^/CD38^−^	After second induction	Both neg: 3-year OS 66%, CIR 35% MRDpos and LSCneg: OS 68%, CIR 43% MRDneg and LSCpos: OS 53%, CIR 53% Both pos: OS 0%, CIR 10%
[53]	Novel leukemia-specific markers	CD9, CD18, CD25, CD32, CD44, CD47, CD52, CD54, CD59, CD64, CD68, CD86, CD93, CD96, CD97, CD99, CD123, CD200, CD300a/c, CD366, CD371, CX3CR1	At diagnosis (to identify novel markers), compared to healthy donors and relapsed AML patients.	Improved sensitivity (1 in 10^5^) Clinical outcomes yet to be studied

LAIP = leukaemia-associated immunophenotype; RFS = relapse-free survival; qPCR = real time quantitative polymerase chain reaction; OS = overall survival; DFS = disease-free survival; NGS = next generation sequencing; DfN = different from normal; alloSCT = allogeneic stem cell transplant; LSC = leukaemic stem cells; CIR = cumulative incidence of relapse; AML = acute myeloid leukaemia.
